# Effectiveness of plasma atherogenic index as a predictor of cardiovascular risk in patients with metabolic syndrome and related cardiometabolic conditions: a systematic review

**DOI:** 10.1097/XCE.0000000000000361

**Published:** 2026-07-01

**Authors:** Mohammed Essam Shaybah, Ghezlan Talal Alenezi, Mohammed Mubarak Alqahtani, Abdulmajeed Saeed Yamaniey, Hasan Hussain Al Farhan, Abdullah Kamal Muslmani, Nada Mohammed Alghamdi, Leen Essam Almahmoud, Juman Omar Alammar, Reem Salem Bajaman, Hanin Saleh Alhawas, Rahaf Jaber Alnefaie, Thamer Alharthy, Turky Abdullah Alharthy, Hussam Ehmid Abu Mousa

**Affiliations:** a College of Medicine, Umm Al-Qura University, Makkah; b College of Medicine, Kuwait University, Kuwait City, Kuwait; c College of Medicine, Prince Sattam bin Abdulaziz University, Al-Kharj; d College of Medicine, King Saud bin Abdulaziz University for Health Sciences, Jeddah; e College of Medicine, Sinnar University, Sudan; f College of Medicine, Taibah University, Medina; g College of Medicine, Qassim University, Buraydah; h College of Medicine, Alfaisal University, Riyadh; i Faculty of Medicine, King Abdulaziz University; j College of Medicine, Ibn Sina National College for Medical Studies, Jeddah; k Consultant in Internal Medicine and Head Department of Internal Medicine, Kingdom Medicine Medical Center, Mecca, Saudi Arabia

**Keywords:** Atherogenic index of plasma, cardiovascular risk, dyslipidemia, major adverse cardiovascular events, metabolic syndrome

## Abstract

Metabolic syndrome (MetS) and cardiovascular–kidney-metabolic syndrome represent escalating global health challenges characterized by complex lipid derangements. While traditional lipid panels are standard, the atherogenic index of plasma (AIP) has emerged as a potentially superior marker for capturing the presence of small, dense low-density lipoprotein particles and overall cardiovascular risk. This systematic review evaluates the prognostic value and longitudinal risk prediction of AIP in patients with MetS and related metabolic disorders. Following Preferred Reporting Items for Systematic Reviews and Meta-Analyses guidelines, a systematic search was conducted across PubMed/MEDLINE, Scopus, Embase, and Web of Science through January 2026. Nine high-quality studies encompassing 197 279 patients were identified. Synthesis of the evidence consistently demonstrates that elevated baseline AIP is consistently and independently associated with major adverse cardiovascular events (hazard ratio: 1.07–1.19), acute myocardial infarction, and cardiovascular death. Longitudinal data reveal that cumulative AIP exposure significantly increases stroke risk (hazard ratio: 2.49) and coronary heart disease. Notably, in patients with diabetic kidney disease, a nonlinear ‘U-shaped’ association was identified, with a critical inflection point at 0.14, suggesting that both excessively high and low AIP levels correlate with increased mortality. AIP is an accessible and promising biomarker that warrants further comparative validation in predicting long-term cardiometabolic progression. These findings support further evaluation of AIP’s role in clinical risk stratification, pending prospective validation and establishment of standardized thresholds across population subgroups.

## Introduction

Metabolic syndrome (MetS) has emerged as a predominant global health challenge, characterized by a complex constellation of physiological, biochemical, and metabolic abnormalities that significantly elevate the risk of cardiovascular disease (CVD) and type 2 diabetes mellitus (T2DM) [1,2]. The fundamental components of MetS, including central obesity, hypertension, insulin resistance, and atherogenic dyslipidemia, act synergistically to promote a proinflammatory and prothrombotic state [3,4]. Despite the implementation of various diagnostic frameworks by organizations such as the International Diabetes Federation and the National Cholesterol Education Program, the rising prevalence of obesity and sedentary lifestyles continues to drive a global epidemic of MetS across both developed and developing nations.

A critical feature of MetS-related cardiovascular risk is atherogenic dyslipidemia, typically manifested as elevated serum triglycerides (TG), low levels of high-density lipoprotein cholesterol (HDL-C), and the predominance of small, dense low-density lipoprotein particles [5]. Recent longitudinal evidence suggests that atherogenic index of plasma (AIP) may provide complementary predictive value alongside traditional lipid ratios in predicting long-term cardiovascular mortality and new-onset metabolic disorders, including metabolic dysfunction associated with fatty liver disease and subclinical hypothyroidism [6–8].

Despite the growing body of literature highlighting the utility of AIP, its specific diagnostic accuracy and consistency in the context of MetS remain subjects of ongoing debate. Prior studies have reported varying results concerning the optimal AIP thresholds, often hindered by small sample sizes, regional population differences, and heterogeneous diagnostic criteria for MetS [9,10]. Furthermore, while some research emphasizes AIP’s role in identifying individuals with subclinical myocardial injury, other studies suggest its predictive power may be mediated by existing metabolic comorbidities [11]. These inconsistencies underscore the necessity for a rigorous synthesis of evidence to clarify the relationship between AIP and MetS across diverse cohorts.

While MetS served as the primary index condition for this review, the scope was extended to encompass closely related cardiometabolic conditions including T2DM, cardiovascular–kidney–metabolic (CKM) syndrome, diabetic kidney disease (DKD), and populations with abnormal glucose metabolism, because these conditions share the same pathophysiological substrate of atherogenic dyslipidemia and represents a clinical spectrum along which AIP may serve as a prognostic biomarker.

The objective of our systematic review is to comprehensively evaluate the prognostic value and longitudinal risk prediction of the AIP in patients with MetS and related cardiometabolic conditions, including T2DM, CKM syndrome, and DKD. By adhering to the Preferred Reporting Items for Systematic Reviews and Meta-Analyses (PRISMA) guidelines, this study seeks to consolidate data from high-quality observational research, assess methodological rigor using the Newcastle–Ottawa Scale (NOS), and determine whether AIP can serve as a standardized, cost-effective tool for identifying individuals at the highest risk of cardiometabolic progression.

## Methodology

### Study guidelines and protocol

Our systematic review was designed, conducted, and reported in strict accordance with the PRISMA 2020 statement [12]. A comprehensive methodological protocol was developed before commencing the review to ensure transparency, rigor, and reproducibility at each stage, encompassing database searching, study selection, data extraction, and quality appraisal. This review was not prospectively registered in PROSPERO or another publicly accessible registry; this is acknowledged as a limitation. Two independent reviewers performed all screening and extraction stages, with discrepancies resolved by consensus or through arbitration by a third reviewer.

A structured and comprehensive literature search was independently executed across four major electronic databases, PubMed/MEDLINE, Scopus, Embase, Web of Science (WOS), covering all records published from database inception through January 2026, without date restrictions on earlier literature. The search strategy was constructed using a combination of medical subject headings (MeSH) terms and free-text keywords, linked by Boolean operators (AND/OR), to capture all relevant dimensions of the research question. For PubMed, the primary search string was: (‘Metabolic Syndrome’ [MeSH Terms] OR ‘Metabolic Syndrome’ [All Fields] OR ‘insulin resistance’ [All Fields] OR ‘cardiometabolic syndrome’ [All Fields]) AND (‘Atherogenic Index of Plasma’ [All Fields] OR ‘AIP’ [All Fields] OR ‘log(TG/HDL-C)’ [All Fields]) AND (‘cardiovascular disease’ [MeSH Terms] OR ‘cardiovascular risk’ [All Fields] OR ‘MACE’ [All Fields] OR ‘coronary heart disease’ [All Fields] OR ‘stroke’ [All Fields] OR ‘mortality’ [All Fields]). Equivalent search strings, adapted to the controlled vocabulary of each platform, were applied to the remaining databases (Supplementary Table S1, Supplemental digital content 1, https://links.lww.com/CAEN/A82). No language restrictions were imposed. To further maximize retrieval sensitivity and minimize publication bias, the reference lists of all eligible full-text articles and pertinent review papers were manually screened for additional studies not captured by electronic search. Gray literature, including preprint servers, was also consulted for nonabstract records where appropriate; conference abstracts were excluded from the synthesis as per the eligibility criteria, as they typically lack sufficient methodological detail for quality assessment.

### Study screening process

**Fig. 1:**
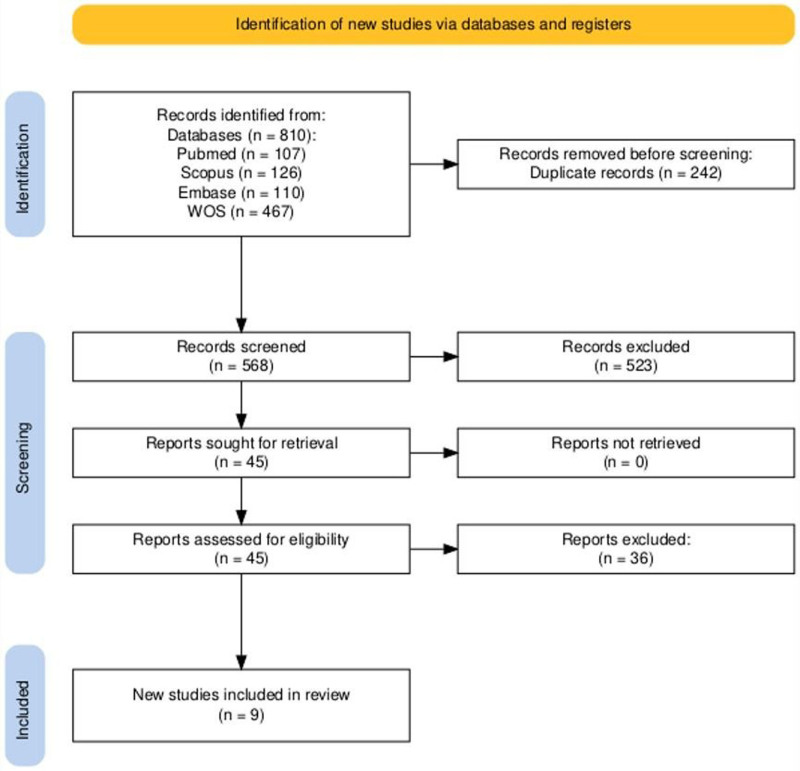
PRISMA 2020 flow diagram illustrating the study selection process. PRISMA, Preferred Reporting Items for Systematic Reviews and Meta-Analyses; WOS, Web of Science.

The screening and selection of studies were conducted using Rayyan, a web-based tool designed for systematic review [13,14] by two independent reviewers across two sequential stages, adhering strictly to the predefined eligibility criteria. In the first stage, all retrieved records were evaluated at the level of title and abstract. Citations were excluded at this stage if they clearly did not fulfill the inclusion criteria based on study population, exposure, or outcome; in cases of uncertainty, the full text was retrieved for further evaluation. In the second stage, the full texts of all potentially eligible articles were independently assessed by both reviewers against the complete Population, Intervention, Comparator, and Outcome (PICO)-based inclusion and exclusion criteria. Disagreements at either screening stage were discussed and resolved by consensus; where consensus could not be reached, a third senior reviewer served as arbiter. The reasons for the exclusion of full-text articles were documented for each rejected study. All screening and selection activities were managed using a standardized screening form. The entire selection process was recorded and reported in accordance with the PRISMA 2020 flow diagram, which is presented in Fig. 1.

### Selection criteria

The eligibility of studies was defined according to the PICO framework as follows:

#### Population:

Adult participants (age ≥18 years) diagnosed with MetS or its constituent clinical components, including T2DM, prediabetes, and central obesity.

#### Exposure:

The AIP is defined as the logarithmically transformed ratio of molar concentrations of triglycerides to HDL-C. This included baseline measurements, longitudinal trajectories, or cumulative exposure [cumulative AIP (CumAIP)].

#### Comparator:

Low AIP levels or the reference group representing the lowest categorized AIP distribution (e.g. the lowest tertile or quartile).

#### Outcome:

Incidence of major adverse cardiovascular events (MACE), specifically encompassing acute myocardial infarction, stroke, cardiovascular death (CVD), heart failure, and unstable angina.

Primary research studies, including prospective and retrospective cohort studies, as well as secondary analyses of randomized controlled trials (RCTs), were eligible for inclusion. No language restrictions were applied. We excluded animal- or laboratory-based models; case reports, series, letters, conference abstracts, and editorials; and secondary research such as existing systematic reviews or meta-analyses.

### Data extraction

Data were extracted independently by two reviewers using a prepiloted, standardized extraction form. Any disagreements were adjudicated by a third reviewer until consensus was reached. The following variables were systematically collected from each eligible study: (a) study identification details, including first author, publication year, and country of origin; (b) study design and the recruitment period; (c) participant characteristics, including sample size, mean age, sex distribution, and relevant comorbidities; (d) definition criteria applied for MetS and related metabolic phenotypes; (e) AIP measurement methodology, including the formula used [log(TG/HDL-C) in molar concentrations], categorization approach (e.g. tertiles, quartiles, or trajectory groups), and baseline vs. cumulative exposure metrics; (f) follow-up duration; (g) primary and secondary outcome definitions; and (h) fully adjusted effect estimates [hazard ratios or odds ratios (ORs)] with corresponding 95% confidence intervals (CIs) and the covariates included in the multivariable models.

### Quality assessment

The risk of bias of all included studies was independently appraised by two reviewers using the NOS, an instrument validated for the critical appraisal of nonrandomized observational studies in systematic reviews. The NOS evaluates three broad domains: (a) selection of the study population (maximum four stars), assessing the representativeness of the exposed cohort, selection of the nonexposed cohort, ascertainment of the exposure, and demonstration that the outcome of interest was not present at study onset; (b) comparability of cohorts based on the design or analysis (maximum two stars), evaluating whether the most important confounders were controlled for in the statistical models; and (c) assessment of outcome or exposure (maximum three stars), examining the adequacy of outcome ascertainment, the sufficiency of follow-up duration, and the completeness of follow-up data. Studies achieving a cumulative NOS score of seven stars or above were classified as high quality and low risk of bias; scores of 4–6 stars were considered moderate quality; and scores below four stars were deemed low quality. For the secondary analysis of the RCT (ACCORD study), the Cochrane Risk of Bias Tool 2 criteria were additionally considered to contextualize potential limitations inherent in secondary data analyses.

### Data synthesis

Given the marked clinical and methodological heterogeneity across the included studies – encompassing differences in study design, population characteristics, AIP categorization approaches, follow-up durations, covariate adjustment strategies, and outcome definitions – formal quantitative pooling via meta-analysis was not undertaken, as this would risk producing misleading summary estimates. Findings were grouped thematically according to: (a) the association between baseline AIP and incident MACE and coronary outcomes; (b) the relationship between CumAIP exposure and cerebrovascular risk; (c) the role of long-term AIP control trajectories on CVD incidence; (d) AIP as a prognostic biomarker within the CKM syndrome spectrum; and (e) threshold and nonlinear effects of AIP in DKD. For each outcome domain, effect estimates (hazard ratio or OR) with 95% CIs were tabulated and narratively compared across studies, with attention to the direction, magnitude, and statistical significance of associations. Heterogeneity was qualitatively assessed by examining overlapping CIs and the consistency of findings across populations.

## Results

### Study selection and data extraction

The electronic database search across PubMed (*n* = 107), Scopus (*n* = 126), Embase (*n* = 110), and WOS (*n* = 467) yielded a total of 810 records. Following the removal of 242 duplicate records, 568 unique records underwent initial screening, of which 523 were excluded. The remaining 45 reports were sought for retrieval, and all 45 were successfully retrieved and assessed for eligibility. During the full-text assessment, 36 reports were subsequently excluded. Ultimately, nine new studies fulfilled all inclusion criteria and were included in the review. The complete screening and selection process is detailed in the PRISMA 2020 flow diagram (Fig. 1).

### Risk of bias

The methodological quality of all nine included studies was independently evaluated using the NOS. Overall, the included body of evidence demonstrated high methodological quality. Four studies achieved the maximum NOS score of 9/9 stars, and five studies attained a score of 8/9 stars (Table 1). However, it is important to note that high NOS scores reflect structural quality and do not fully eliminate concerns regarding residual confounding, differences in exposure categorization, or outcome heterogeneity across cohorts. The selection domain scores reflect well-defined, representative cohorts with appropriate ascertainment of AIP and confirmation of outcome-free status at baseline. Comparability scores were generally strong; the most commonly adjusted confounders included age, sex, BMI, glycemic parameters (HbA1c or fasting glucose), blood pressure, and lipid-lowering medication use. However, adjustment for smoking status, antihypertensive therapy, baseline CVD, and kidney function (estimated glomerular filtration rate or chronic kidney disease stage) was inconsistent across studies, introducing potential residual confounding. Outcome assessment was conducted via linkage to validated national registries or electronic health records in most studies, with follow-up periods sufficient to capture cardiovascular events. Exposure ascertainment varied across studies – some used single baseline AIP measurements, others used CumAIP or trajectory-based approaches – contributing to clinical heterogeneity that limits direct comparison of effect estimates. Table 1 provides an expanded summary of per-study NOS domain scores and the key covariates included in each study’s primary multivariable model.

**Table 1:** Risk of bias assessment

References	Study design	Selection	Comparability	Outcome	Total score	Quality
Lin *et al.* [15]	Longitudinal cohort	★★★★	★★	★★	8/9	High
Zheng *et al.* (mortality) [16]	Prospective cohort	★★★★	★★	★★★	9/9	High
Li and Xu [17]	Prospective (NHANES)	★★★★	★★	★★★	9/9	High
Zheng *et al.* (CVD) [18]	Nationwide cohort	★★★★	★★	★★	8/9	High
Sun *et al*. [19]	Longitudinal cohort	★★★★	★★	★★	8/9	High
Hu *et al*. (UKB) [20]	Prospective cohort	★★★★	★★	★★★	9/9	High
Min *et al*. [21]	Longitudinal cohort	★★★★	★★	★★	8/9	High
Fu *et al*. [22]	Secondary RCT cnalysis	★★★★	★★	★★★	9/9	High
Hu *et al*. [20]	Prospective cohort	★★★★	★★	★★	8/9	High

CVD, cardiovascular disease; NHANES, National Health and Nutrition Examination Survey; RCT, randomized controlled trial; UKB, UK Biobank.

### Study selection and characteristics

After a comprehensive literature search and screening, nine studies were included in this systematic review, totaling 197 279 patients. The synthesized data primarily address the longitudinal and cross-sectional associations between the AIP and adverse clinical outcomes across various metabolic and renal phenotypes (Table 2).

**Table 2:** Characteristics of the included studies

References	Study design	Country	Duration/timeframe	Sample size and population	Study arms	Follow-up period	Short summary	Total/MetS/comparator	Conclusion and clinical significance
Lin *et al*. [15]	Longitudinal cohort study	China	2011–2018	4525 Middle-aged/elderly with CKM stages 0–3	4 K-means clusters based on AIP trajectories	~7 years (Waves 1–4)	Evaluates AIP trajectories and their association with future cardiovascular disease risk	Total: 4525 MetS: All (CKM 0–3) Comparator: Low-AIP stable group	Elevated and fluctuating AIP levels independently predict an increased risk of CVD in middle-aged and elderly individuals at CKM stages 0–3
Zheng *et al*. (mortality) [16]	Prospective cohort study	USA	2005–2018	15 703 Individuals with CKM syndrome	AIP tertiles (T1, T2, T3)	Median ~10 years (to 2019)	Investigates the link between AIP and all-cause/cardiovascular mortality in CKM patients	Total: 15 703 MetS: All (CKM) Comparator: AIP Tertile 1 (Ref)	Higher AIP levels are independently associated with an increased risk of all-cause and cardiovascular mortality in patients with CKM syndrome
Li and Xu [17]	Cross-sectional/prospective study	USA	2001–2018	5108 Patients with T2DM and DKD	AIP quartiles (Q1–Q4)	Long-term NHANES tracking	Found a ‘U-shaped’ association between AIP and CVD mortality in DKD patients	Total: 5108 MetS: T2DM population Comparator: AIP Quartile 1	In patients with DKD, the relationship between AIP and CVD mortality follows a ‘U’-shaped curve. Maintaining the index within a specific target range (0.08–0.13) may provide the greatest survival benefit
Zheng *et al*. (CVD risk) [18]	Prospective nationwide cohort study	China	2011–2018	3429 Individuals with CKM stages 0–3	5 AIP control level classes	~7 years	Assesses the impact of cumulative AIP on the incidence of CVD	Total: 3429 MetS: All (CKM 0–3) Comparator: Class 1 (Best control)	Both baseline and cumulative AIP exposure are significant predictors of new-onset CVD in individuals at CKM stages 0–3
Sun *et al*. [19]	Longitudinal cohort study	China	2011–2018	4674 Middle-aged/elderly with CKM stages 0–3	Cumulative AIP quartiles (Q1–Q4)	~7 years	Analyzes association between cumulative AIP and new-onset stroke risk	Total: 4674 MetS: all (CKM 0–3) comparator: quartile 1	Higher cumulative AIP is significantly linked to an increased risk of incident stroke
Hu *et al*. (2025) (UKB) [20]	Prospective cohort study	UK	2006–2010 (Baseline)	131 736 Individuals with metabolic syndrome	Baseline AIP quartiles (Q1–Q4)	Median ~12–14 years	Evaluates AIP’s predictive value for MACE endpoints specifically within a MetS cohort	Total: 131 736 MetS: 131 736 (100%) comparator: quartile 1	Elevated AIP is an independent risk factor for MACE in individuals with metabolic syndrome. Incorporating AIP into clinical assessments can significantly enhance the identification of high-risk patients beyond traditional lipid markers
Min *et al*. [21]	Longitudinal cohort study	China	2011–2018	2659 Adults with abnormal glucose metabolism	5 Classes of AIP control levels	Mean: 3 years (wave 3–4)	Investigates how AIP control levels affect incident CVD in prediabetic/diabetic patients	Total: 2659 MetS: abnormal glucose comparator: class 1	Persistently high AIP with poor control is a strong predictor of incident CVD among middle-aged and elderly Chinese individuals with abnormal glucose metabolism
Fu *et al*. [22]	Secondary analysis of a randomized trial	North America	2001–2014	10 251 Patients with long-lasting T2DM	High AIP (≥0.34) vs. low AIP (<0.34)	Median: 9.7 years	Identifies AIP as an independent risk factor for MACE and mortality in T2DM patients	Total: 10 251 MetS: T2DM population comparator: low-AIP group	AIP is a powerful and independent prognostic biomarker for MACE, cardiovascular death, and nonfatal myocardial infarction in patients with type 2 diabetes
Hu *et al*. (elderly) [20]	Prospective cohort study	China	2018–2023	19 194 Chinese elderly (60+ years)	AIP trajectory groups (low, medium, high)	Mean: 2.65 years	Focuses on persistent high AIP trajectories and their link to new-onset coronary heart disease.	Total: 19 194 MetS: not explicitly defined comparator: low-level trajectory	A significant correlation exists between persistent high-level AIP trajectories and the risk of new-onset CHD in the elderly

AIP, atherogenic index of plasma; CHD, coronary heart disease; CKM, cardiovascular–kidney–metabolic; CVD, cardiovascular disease; DKD, diabetic kidney disease; MACE, major adverse cardiovascular event; MetS, metabolic syndrome; NHANES, National Health and Nutrition Examination Survey; T2DM, type 2 diabetes mellitus; UKB, UK Biobank.

### Atherogenic index of plasma and major adverse cardiovascular events

The AIP is consistently identified as an independent predictor of MACE and coronary complications across diverse metabolic profiles, with associations that appear robust across heterogeneous study populations. In a large prospective analysis of 131 736 individuals with MetS, Hu *et al*. [23] reported that each SD increase in baseline AIP was significantly associated with a higher risk of MACE (hazard ratio: 1.07, 95% CI: 1.05–1.08), AMI (hazard ratio: 1.16, 95% CI: 1.12–1.19), and unstable angina (hazard ratio: 1.16, 95% CI: 1.13–1.19).

These findings were corroborated by data from the ACCORD study [22] involving 10 251 patients with T2DM, which confirmed AIP as an independent risk factor for MACE (hazard ratio: 1.194, 95% CI: 1.049–1.360), CVD (hazard ratio: 1.264, 95% CI: 1.015–1.573), and nonfatal myocardial infarction (hazard ratio: 1.284, 95% CI: 1.071–1.539).

The relationship between AIP trajectories and coronary health further underscores this risk. Hu *et al*. [20] demonstrated in a longitudinal study of 19 194 elderly individuals that those in the high-level AIP trajectory group (mean AIP: 0.38–0.42) faced a 43% higher risk of new-onset coronary heart disease (CHD) compared with those in low-level trajectory groups (hazard ratio: 1.43, 95% CI: 1.19–1.73) (Table 3).

**Table 3:** Summary of the results

References	Population (*N*) and condition	AIP measurement	Primary cardiovascular outcome(s)	Results (HR/OR, 95% CI)
Hu *et al*. [23]	131 736 (Metabolic syndrome)	Baseline	MACE (composite) Acute MI Unstable angina	HR: 1.07 (1.05–1.08) per SD increase HR: 1.16 (1.12–1.19) HR: 1.16 (1.13–1.19)
Hu *et al*. [20]	19 194 (Elderly Chinese)	Trajectory (2018–2020)	New-onset CHD	HR: 1.43 (1.19–1.73) (high vs. low trajectory)
Zheng *et al*. [16]	15 703 (CKM syndrome)	Tertiles (baseline)	CV mortality All-cause mortality	HR: 1.38 (1.22–1.57) (T3 vs. T1) HR: 1.19 (1.08–1.31) (T3 vs. T1)
Fu *et al*. [22]	10 251 (Type 2 diabetes)	Threshold (>0.34)	MACE (composite) CV death Nonfatal MI	HR: 1.194 (1.049–1.360) HR: 1.264 (1.015–1.573) HR: 1.284 (1.071–1.539)
Sun *et al*. [19]	4674 (CKM stages 0–3)	Cumulative (CumAIP)	New-onset stroke	HR: 2.49 (1.69–3.65) per unit increase
Lin *et al*. [15]	4525 (CKM stages 0–3)	Quartiles (CumAIP)	Incident CVD	HR: 1.58 (1.14–2.19) (Q4 vs. Q1)
Zheng *et al*. [18]	3429 (CKM stages 0–3)	Cluster (control level)	Incident CVD	OR: 2.14 (1.15–3.97) (worst vs. best control)
Min *et al*. [21]	2659 (Abnormal glucose)	Cluster (control level)	Incident CVD	OR: 1.56 (1.03–2.37) (worst vs. best control)
Li and Xu [17]	1598 (Diabetic kidney disease)	Threshold/RCS	CV mortality	HR: 1.68 (1.15–2.45) (AIP ≥ 0.14) HR: 0.62 (0.40–0.96) (AIP < 0.14)

AIP, atherogenic index of plasma; CHD, coronary heart disease; CI, confidence interval; CKM, cardiovascular–kidney–metabolic; CV, cardiovascular; CVD, cardiovascular disease; HR, hazard ratio; MACE, major adverse cardiovascular event; MI, myocardial infarction; OR, odds ratio; RCS, radar cross-section.

### Cumulative exposure and stroke risk

The impact of AIP on cerebrovascular outcomes is particularly evident when considering cumulative exposure over time. Sun *et al*. [19] found that among 4674 middle-aged and elderly Chinese patients, each unit increase in CumAIP was associated with a 149% higher risk of incident stroke (hazard ratio: 2.49, 95% CI: 1.69–3.65). Analysis from the CHARLS cohort suggests that these cardiovascular risks are closely linked to the lipid profile, where triglycerides serve as the primary driver for AIP in predicting future CVD events [16].

Long-term AIP control levels also significantly influence clinical outcomes. Min *et al*. [21] observed in 2659 participants that those with consistently high AIP levels, representing the poorest control, exhibited a 1.56-fold higher risk of incident CVD (OR: 1.56, 95% CI: 1.03–2.37) compared with the best-controlled group. Supporting this, longitudinal monitoring of 4525 participants by Lin *et al*. [15] revealed a clear ascending trend in CVD risk across CumAIP quartiles (*P* for trend = 0.014).

### Atherogenic index of plasma in cardiovascular–kidney–metabolic syndrome

AIP serves as a critical biomarker for risk stratification within the spectrum of CKM syndrome and renal impairment. Zheng *et al*. [18] analyzed 15 703 National Health and Nutrition Examination Survey participants and demonstrated that individuals in the highest AIP tertile faced significantly elevated risks of all-cause mortality (hazard ratio: 1.19, 95% CI: 1.08–1.31) and cardiovascular mortality (hazard ratio: 1.38, 95% CI: 1.22–1.57). This association with all-cause mortality was particularly significant in advanced CKM stages (stages 3–4), where the risk increased 1.17-fold for every SD increase in AIP.

Furthermore, poor long-term AIP control in earlier CKM stages (0–3) was associated with more than a twofold risk of developing CVD compared to those with the best control (OR: 2.14, 95% CI: 1.15–3.97), a finding further supported by the cumulative risk trends identified by Lin *et al*. [15] and Zheng *et al*. [16].

### Threshold effects on diabetic kidney disease

In the specific context of DKD, Li and Xu [17] identified a nonlinear, ‘U’-shaped association between AIP and CVD mortality. Threshold effect analysis identified a critical inflection point at an AIP value of 0.14. When AIP was below this threshold (AIP < 0.14), there was a significant decrease in CVD mortality risk (hazard ratio: 0.62, 95% CI: 0.40–0.96, *P* = 0.033). Conversely, when AIP was at or above this threshold (AIP ≥ 0.14), there was a marked increase in CVD mortality risk (hazard ratio: 1.68, 95% CI: 1.15–2.45, *P* = 0.008). These results highlight the importance of maintaining AIP within a specific narrow range to mitigate mortality risk in patients with renal impairment.

## Discussion

### Summary of the key findings

Our systematic review, encompassing a substantial cohort of 197 279 patients across nine studies, synthesizes evidence on the consistent association between the AIP and adverse clinical outcomes across diverse metabolic and renal phenotypes. Our synthesis consistently suggests that AIP is independently associated with MACE and coronary complications, particularly within populations grappling with MetS, T2DM, and among elderly individuals [20,22,23]. Crucially, the analysis reveals that not only baseline AIP but also CumAIP exposure and the long-term control level of AIP significantly modulate the risk of stroke and incident CVD [15,16,19,21]. Furthermore, AIP emerges as a critical biomarker for risk stratification within the complex spectrum of CKM syndrome, demonstrating significant associations with all-cause and cardiovascular mortality, especially in advanced CKM stages [18]. A particularly noteworthy finding is the nonlinear, ‘U’-shaped association identified between AIP and CVD mortality in patients with DKD, highlighting a specific critical threshold that dictates mortality risk [17].

### Explanation of the study results

The consistent identification of AIP as an independent risk factor for MACE and coronary complications across various metabolic profiles underscores its utility as a comprehensive indicator of lipid-related cardiovascular risk. AIP, calculated as the logarithmic ratio of triglycerides to HDL-C [log(TG/HDL-C)], intrinsically reflects the balance between atherogenic and antiatherogenic lipoproteins [24]. Dyslipidemia, characterized by elevated triglycerides and reduced HDL-C, is a well-established driver of atherosclerotic progression and is central to the pathophysiology of MetS and T2DM [25,26]. Our findings, such as those demonstrating a 1.07-fold higher risk of MACE for each SD increase in AIP in MetS patients [23], align with the established understanding that an unfavorable lipid profile promotes endothelial dysfunction, inflammation, and plaque formation [27]. Similarly, the corroboration of AIP as an independent risk factor for MACE, CVD, and nonfatal myocardial infarction in T2DM patients [22] emphasizes its relevance in a population already at high cardiovascular risk because of complex metabolic derangements, as has been documented across multiple diabetic complication domains [28].

The impact of AIP trajectories and cumulative exposure further elucidates the chronic burden of dyslipidemia on vascular health. The observation that consistently high AIP levels significantly elevate the risk of new-onset CHD [20] and incident CVD [15,21] suggests that sustained exposure to an atherogenic lipid environment promotes progressive vascular damage [29].

This is powerfully demonstrated by the finding that each unit increase in CumAIP substantially elevates the risk of incident stroke [19], reinforcing the concept that long-term metabolic control, rather than sporadic measurements, is paramount in preventing cerebrovascular events [30]. This principle of cumulative metabolic damage extends beyond cardiovascular outcomes. Chen *et al*. [31] demonstrated that key risk factors for cataract development in diabetic patients included high HbA1c levels and longer diabetes duration, further underscoring that sustained hyperglycemia drives multiorgan pathology, including ocular complications. The role of triglycerides as the primary driver for AIP’s predictive power in future CVD events further highlights the importance of managing this specific lipid component [16]. These lipid-centric findings complement the cardiovascular and renal protective benefits observed with SGLT2 inhibitors, which have been shown to reduce nonfatal myocardial infarction by 12%, hospitalization for heart failure by 33%, and cardiac death by 15% in patients with T2DM [32]

AIP’s critical role in CKM syndrome risk stratification reflects the intertwined nature of cardiovascular, kidney, and metabolic dysfunctions. CKM syndrome represents a spectrum of progressive multiorgan disease, where dyslipidemia often contributes to the pathology of all three components [33]. Pharmacologically targeting this dyslipidemia confers multiorgan benefits. Chen *et al*. [34] found that fibrate therapy not only reduced diabetic retinopathy incidence and progression but also demonstrated a favorable safety profile, with no significant increase in all-cause mortality compared to placebo (OR: 0.86, 95% CI: 0.62–1.19, *P* = 0.36). Furthermore, combining fibrates with statins reduced diabetic retinopathy progression by an additional 17% compared with fibrates alone (hazard ratio: 0.84, 95% CI: 0.80–0.89, *P* < 0.001). The observed association between higher AIP tertiles and significantly elevated risks of all-cause and cardiovascular mortality, particularly in advanced CKM stages [33], indicates that AIP can serve as a valuable tool for identifying individuals with greater systemic vulnerability. Poor long-term AIP control in earlier CKM stages increases CVD risk and also supports proactive management strategies across the entire CKM continuum [15,16,35].

The ‘U’-shaped association between AIP and CVD mortality in DKD patients [17] represents a significant nuance. This nonlinear relationship, with a critical inflection point at an AIP value of 0.14, suggests that both excessively low and high AIP values may be detrimental, possibly reflecting different underlying pathophysiological mechanisms [36]. This threshold concept parallels findings from Chen *et al*. [37], who demonstrated a dose-dependent protective effect of omega-3 fatty acids against diabetic retinopathy, with a specific threshold of greater than or equal to 500 mg/day producing a 48% risk reduction for sight-threatening disease. Notably, the study emphasized that the greatest benefits came from whole food sources and optimal omega-6/omega-3 ratios, suggesting that both the quantity and quality of nutritional interventions matter – a concept that may extend to AIP optimization, where achieving a specific target range appears critical.

While high AIP clearly indicates dyslipidemia and increased atherosclerosis, an extremely low AIP might, in some contexts, be associated with other metabolic disturbances or severe malnutrition, although this warrants further investigation [38–40]. This finding challenges a purely linear interpretation of AIP and underscores the need for precise individualized management in this vulnerable population.

Our systematic review contributes significantly to the existing literature by synthesizing a large body of evidence from a large number of patients, offering a comprehensive and unified perspective on AIP’s prognostic value across a broad spectrum of metabolic and renal conditions. While individual studies have explored aspects of AIP, our work distinguishes itself by systematically integrating findings on longitudinal associations, the impact of CumAIP exposure, and the significance of long-term AIP control levels, which collectively offer a more dynamic understanding of its role in disease progression [41,42]. The emphasis on diverse populations, including those with MetS, T2DM, the elderly, and various CKM stages, broadens the applicability of AIP as a universal risk marker. Furthermore, the identification and careful characterization of the nonlinear, ‘U’-shaped association between AIP and CVD mortality specifically within the context of DKD is a novel contribution that refines our understanding of optimal lipid management targets in this high-risk group. This systematic approach provides a robust evidence base, moving beyond isolated observations to offer a holistic view of AIP’s critical role in cardiovascular–kidney metabolic health.

### Strengths and limitations

Our systematic review benefits from several key strengths. The inclusion of a large patient cohort (197 279 individuals) drawn from nine studies significantly enhances the statistical power and generalizability of our findings. The systematic methodology ensured a comprehensive evaluation of the literature, reducing potential selection bias. A particular strength lies in the focus on longitudinal data and the investigation of CumAIP exposure and long-term control levels, which provide a more accurate reflection of chronic disease processes compared with single-point measurements. The analysis also spanned diverse and clinically relevant populations, including those with MetS, T2DM, elderly individuals, and various stages of CKM syndrome, reinforcing the broad applicability of AIP. The identification of a specific threshold effect in DKD represents an important clinical insight. Despite these strengths, several limitations must be acknowledged. A key methodological limitation is that this review was not prospectively registered in PROSPERO or another systematic review registry, which limits the verifiability of the a priori protocol. First, the reliance on observational studies, inherent to systematic reviews of this nature, precludes definitive conclusions about causality. While strong associations were observed, residual confounding factors, despite adjustments in the original studies, cannot be entirely ruled out. Second, heterogeneity across the included studies regarding population characteristics, follow-up durations, and specific methods for AIP calculation or outcome assessment could introduce variability in findings. Thirdly, the review primarily focuses on the predictive value of AIP and does not include interventional trials; thus, it cannot provide direct guidance on therapeutic interventions targeting AIP to improve outcomes. The mechanisms underlying the ‘U’-shaped association in DKD, while identified, require further mechanistic exploration and validation through dedicated prospective studies. Finally, the generalizability of findings to certain ethnic groups or younger populations not extensively represented in the included studies remains to be fully explored.

### Implications, clinical impact, and future directions

The implications of our findings are substantial, suggesting that AIP is a readily available and highly informative biomarker that can significantly enhance cardiovascular risk stratification beyond conventional lipid panels. Its consistent association with MACE, stroke, and mortality across various metabolic and renal conditions positions it as a vital component in a holistic risk assessment strategy. The clinical impact of these findings is profound. Integrating AIP into routine clinical practice, particularly for individuals with MetS, T2DM, CKM syndrome, or the elderly, could refine risk prediction and facilitate more personalized prevention strategies. Given its ease of calculation from standard lipid profiles, AIP offers a cost-effective and accessible tool for identifying high-risk individuals who may benefit from intensified lifestyle modifications or pharmacotherapy. The emphasis on long-term AIP control highlights the importance of sustained metabolic management rather than transient improvement. For DKD patients, the identified ‘U’-shaped association and critical threshold of 0.14 for AIP underscore the need for careful monitoring and potentially tailored interventions to maintain AIP within an optimal range, thereby mitigating CVD mortality risk. Future research should prioritize prospective interventional studies to conclusively determine whether therapeutic strategies aimed at modifying AIP levels – including lipid-lowering agents such as fibrates, which have demonstrated vascular protective effects in diabetes-related dyslipidemia, and omega-3 fatty acid supplementation, which has shown favorable effects on triglyceride-mediated atherogenic indices – can lead to improved clinical outcomes. Mechanistic studies are warranted to fully elucidate the biological pathways through which AIP exerts its predictive power, particularly concerning the observed nonlinear relationship in DKD. Further research is also needed to establish optimal AIP thresholds for various populations and disease states and to explore its interaction with genetic predispositions and other circulating biomarkers. Investigation into the cost-effectiveness of AIP-guided interventions and their utility in other underserved or specific ethnic populations will also be valuable.

### Conclusion

Our comprehensive systematic review demonstrates that the AIP is consistently and independently associated with MACEs, stroke, and all-cause and cardiovascular mortality across the cardio–renal–metabolic disease spectrum, suggesting it may serve as a practical and accessible prognostic marker. The significance of cumulative exposure and long-term control of AIP, coupled with its relevance in CKM syndrome and the nuanced ‘U’-shaped association in DKD, underscores its potential clinical utility. However, its incremental value beyond established lipid risk models, the identification of optimal and validated thresholds across diverse populations, and its role in guiding therapeutic interventions require further prospective validation before routine clinical implementation can be universally recommended.

## Acknowledgements

All authors agree to participate in this research study and consent to be published.

### Conflicts of interest

There are no conflicts of interest.

## Supplementary Material

## References

[1] Caballero B. The global epidemic of obesity: an overview. Epidemiol Rev 2007; 29:1–5. 17569676 10.1093/epirev/mxm012

[2] Dobrowolski P Prejbisz A Kuryłowicz A Baska A Burchardt P Chlebus K. Metabolic syndrome – a new definition and management guidelines: a joint position paper by the Polish Society of Hypertension, Polish Society for the Treatment of Obesity, Polish Lipid Association, Polish Association for Study of Liver, Polish Society of Family Medicine, Polish Society of Lifestyle Medicine, Division of Prevention and Epidemiology Polish Cardiac Society, ‘Club 30’ Polish Cardiac Society, and Division of Metabolic and Bariatric Surgery Society of Polish Surgeons. Arch Med Sci 2022; 18:1133–1156. 36160355 10.5114/aoms/152921 PMC9479724

[3] Jia G Hill MA Sowers JR. Vascular endothelial mineralocorticoid receptors and epithelial sodium channels in metabolic syndrome and related cardiovascular disease. J Mol Endocrinol 2023; 71:1. 10.1530/JME-23-0066 PMC10502958 37610001

[4] Jung UJ Choi M-S. Obesity and its metabolic complications: the role of adipokines and the relationship between obesity, inflammation, insulin resistance, dyslipidemia and nonalcoholic fatty liver disease. Int J Mol Sci 2014; 15:6184–6223. 24733068 10.3390/ijms15046184 PMC4013623

[5] Dobiásová M Frohlich J. The plasma parameter log (TG/HDL-C) as an atherogenic index: correlation with lipoprotein particle size and esterification rate in apoB-lipoprotein-depleted plasma (FER(HDL)). Clin Biochem 2001; 34:583–588. 11738396 10.1016/s0009-9120(01)00263-6

[6] Dobiásová M Urbanová Z Samánek M. Relations between particle size of HDL and LDL lipoproteins and cholesterol esterification rate. Physiol Res 2005; 54:159–165. 15544423

[7] Li Y-W Kao T-W Chang P-K Chen W-L Wu L-W. Atherogenic index of plasma as predictors for metabolic syndrome, hypertension and diabetes mellitus in Taiwan citizens: a 9-year longitudinal study. Sci Rep 2021; 11:9900. 33972652 10.1038/s41598-021-89307-z PMC8110777

[8] Mao G Chen M Huang L Mo Z Su D Gu S. Differences in vitamin A levels and their association with the atherogenic index of plasma and subclinical hypothyroidism in adults: a cross-sectional analysis in China. Nutrients 2024; 16:2613. 39203751 10.3390/nu16162613 PMC11357057

[9] Zhang X-H Zhang M He J Yan Y-Z Ma J-L Wang K. Comparison of anthropometric and atherogenic indices as screening tools of metabolic syndrome in the Kazakh adult population in Xinjiang. Int J Environ Res Public Health 2016; 13:428. 27092520 10.3390/ijerph13040428 PMC4847090

[10] Andraschko LM Gazi G Leucuta D-C Popa S-L Chis BA Ismaiel A. Atherogenic index of plasma in metabolic syndrome – a systematic review and meta-analysis. Medicina (Kaunas) 2025; 61:611. 40282902 10.3390/medicina61040611 PMC12028871

[11] Sandesara UB Kazibwe R Yeboah J Soliman EZ. Interrelations between atherogenic index of plasma, subclinical myocardial injury, and cardiovascular mortality in the general population. medRxiv 2026.

[12] Hutton B Salanti G Caldwell DM Chaimani A Schmid CH Cameron C. The PRISMA extension statement for reporting of systematic reviews incorporating network meta-analyses of health care interventions: checklist and explanations. Ann Intern Med 2015; 162:777–784. 26030634 10.7326/M14-2385

[13] Ouzzani M Hammady H Fedorowicz Z Elmagarmid A. Rayyan – a web and mobile app for systematic reviews. Syst Rev 2016; 5:210. 27919275 10.1186/s13643-016-0384-4 PMC5139140

[14] Rayyan. Intelligent Systematic Review – Rayyan. 2024. https://www.rayyan.ai/. [Accessed 16 February 2026]

[15] Lin Y Lv X Shi C Wang T Jin Z Jin Q Gu C. Association between atherogenic index of plasma and future cardiovascular disease risk in middle-aged and elderly individuals with cardiovascular-kidney-metabolic syndrome stage 0-3. Front Endocrinol (Lausanne) 2025; 16:1540241. 40162318 10.3389/fendo.2025.1540241 PMC11949822

[16] Zheng G Jin J Wang F Zheng Q Shao J Yao J. Association between atherogenic index of plasma and future risk of cardiovascular disease in individuals with cardiovascular-kidney-metabolic syndrome stages 0-3: a nationwide prospective cohort study. Cardiovasc Diabetol 2025; 24:22. 39827127 10.1186/s12933-025-02589-9 PMC11743013

[17] Li Z Xu H. Association of atherogenic index of plasma with cardiovascular disease mortality in patients with type 2 diabetes mellitus and diabetic kidney disease: a cross-sectional study. BMC Cardiovasc Disord 2025; 25:753. 41120908 10.1186/s12872-025-05234-1 PMC12542293

[18] Zheng Q Cao Z Teng J Lu Q Huang P Zhou J. Association between atherogenic index of plasma with all-cause and cardiovascular mortality in individuals with cardiovascular-kidney-metabolic syndrome. Cardiovasc Diabetol 2025; 24:183. 40287685 10.1186/s12933-025-02742-4 PMC12034140

[19] Sun M Yang Q Zhang R Zhang X Xu L Pan P. Association between cumulative atherogenic index of plasma and new-onset stroke among middle-aged and elderly Chinese patients with stages 0-3 cardiovascular-kidney-metabolic syndrome: a longitudinal cohort study. Brain Behav. 2025; 15:e70914. 40988399 10.1002/brb3.70914 PMC12457722

[20] Hu W-L Cheng Y-L Su D-H Cui Y-F Li Z-H Li G-F. Association between atherogenic index of plasma trajectory and new-onset coronary heart disease in Chinese elderly people: a prospective cohort study. J Geriatr Cardiol 2025; 22:835–843. 41179251 10.26599/1671-5411.2025.10.001 PMC12576797

[21] Min Q Wu Z Yao J Wang S Duan L Liu S. Association between atherogenic index of plasma control level and incident cardiovascular disease in middle-aged and elderly Chinese individuals with abnormal glucose metabolism. Cardiovasc Diabetol 2024; 23:54. 38331798 10.1186/s12933-024-02144-y PMC10854096

[22] Fu L Zhou Y Sun J Zhu Z Xing Z Zhou S. Atherogenic index of plasma is associated with major adverse cardiovascular events in patients with type 2 diabetes mellitus. Cardiovasc Diabetol 2021; 20:201. 34610830 10.1186/s12933-021-01393-5 PMC8493717

[23] Hu Y Li W Nie J Zhang C Zhou J Hu Z. Association between the atherogenic index of plasma and major adverse cardiovascular events in individuals with metabolic syndrome: findings from the UK Biobank. Cardiovasc Diabetol 2025; 24:444. 41272658 10.1186/s12933-025-03010-1 PMC12639847

[24] Dobiásová M Frohlich J Sedová M Cheung MC Brown BG. Cholesterol esterification and atherogenic index of plasma correlate with lipoprotein size and findings on coronary angiography. J Lipid Res 2011; 52:566–571. 21224290 10.1194/jlr.P011668 PMC3035693

[25] Grundy SM. Hypertriglyceridemia, insulin resistance, and the metabolic syndrome. Am J Cardiol 1999; 83:25F–29F. 10.1016/s0002-9149(99)00211-8 10357572

[26] Reaven GM. Role of insulin resistance in human disease (syndrome X): an expanded definition. Annu Rev Med 1993; 44:121–131. 8476236 10.1146/annurev.me.44.020193.001005

[27] Libby P Ridker PM Maseri A. Inflammation and atherosclerosis. Circulation 2002; 105:1135–1143. 11877368 10.1161/hc0902.104353

[28] Fox CS Coady S Sorlie PD D’Agostino RB Pencina MJ Vasan RS. Increasing cardiovascular disease burden due to diabetes mellitus: the Framingham Heart Study. Circulation 2007; 115:1544–1550. 17353438 10.1161/CIRCULATIONAHA.106.658948

[29] Ridker PM Silvertown JD. Inflammation, C-reactive protein, and atherothrombosis. J Periodontol 2008; 79:1544–1551. 18673009 10.1902/jop.2008.080249

[30] Seshadri S Beiser A Kelly-Hayes M Kase CS Au R Kannel WB Wolf PA. The lifetime risk of stroke: estimates from the Framingham Study. Stroke 2006; 37:345–350. 16397184 10.1161/01.STR.0000199613.38911.b2

[31] Chen K-Y Chan H-C Chan C-M. How does diabetes shape the landscape of cataract development and surgical success? A systematic review and meta-analysis. Endocrine 2025; 90:404–419. 40775566 10.1007/s12020-025-04374-w

[32] Liang I-C Chang H-H Lai Y-J Chan C-M Sung C-H Pu C-M. Update on the efficacy and safety of sodium-glucose co-transporter 2 inhibitors in patients with chronic diseases: a systematic review and meta-analysis. Medicina (Kaunas) 2025; 61:202. 40005319 10.3390/medicina61020202 PMC11857657

[33] Ndumele CE Rangaswami J Chow SL Neeland IJ Tuttle KR Khan SS; American Heart Association. Cardiovascular-kidney-metabolic health: a presidential advisory from the American Heart Association. Circulation 2023; 148:1606–1635. 37807924 10.1161/CIR.0000000000001184

[34] Chen K-Y Chan H-C Chan C-M. Can fibrate therapy redefine the management of diabetic retinopathy? A comprehensive systematic review and meta-analysis of efficacy and safety. J Diabetes Complications 2025; 39:109178. 41038126 10.1016/j.jdiacomp.2025.109178

[35] Marassi M Fadini GP. The cardio-renal-metabolic connection: a review of the evidence. Cardiovasc Diabetol 2023; 22:195. 37525273 10.1186/s12933-023-01937-x PMC10391899

[36] Wang B Jiang C Qu Y Wang J Yan C Zhang X. Nonlinear association between atherogenic index of plasma and chronic kidney disease: a nationwide cross-sectional study. Lipids Health Dis 2024; 23:312. 39334373 10.1186/s12944-024-02288-6 PMC11429454

[37] Chen K-Y Chan H-C Chan C-M. Association between omega-3 fatty acid intake and risk of diabetic retinopathy: a systematic review and meta-analysis. J Nutr Health Aging 2025; 29:100632. 40987202 10.1016/j.jnha.2025.100632 PMC12492007

[38] Farmer JA. Diabetic dyslipidemia and atherosclerosis: evidence from clinical trials. Curr Diab Rep 2008; 8:71–77. 18367002 10.1007/s11892-008-0013-2

[39] Huo G Tang Y Zhou D. Nonlinear association between atherogenic index of plasma and unstable carotid plaque: a single-center retrospective study. J Cardiovasc Dev Dis 2025; 12:443. 41295369 10.3390/jcdd12110443 PMC12653747

[40] Mo D Zhang P Zhang M Dai H Wang G. Association between the atherogenic index of plasma and incident hypertension across different blood pressure states: a national cohort study. Cardiovasc Diabetol 2025; 24:219. 40399995 10.1186/s12933-025-02775-9 PMC12093804

[41] Wang M Liu S Liu L Wen X Liao Y Liu H. Association between the cumulative exposure to atherogenic index of plasma and risk of cardiometabolic diseases: a prospective cohort study. Endocrine 2025; 89:67–78. 40146498 10.1007/s12020-025-04197-9 PMC12227351

[42] Liu Z Zhang L Wang L Li K Fan F Jia J. The predictive value of cumulative atherogenic index of plasma (AIP) for cardiovascular outcomes: a prospective community-based cohort study. Cardiovasc Diabetol 2024; 23:264. 39026310 10.1186/s12933-024-02350-8 PMC11264486

