# Supplementary material for: Effectiveness of plasma atherogenic index as a predictor of cardiovascular risk in patients with metabolic syndrome and related cardiometabolic conditions: a systematic review
Source: Cardiovasc Endocrinol Metab. 2026 Jul 1;15(3):e00361. doi: 10.1097/XCE.0000000000000361 (PMC13327390; doi:10.1097/XCE.0000000000000361)
Supplement: Supplementary file 1 [file xce-15-e00361-s001.docx]

| **Database** | **Full Search String** |
| --- | --- |
| **PubMed/MEDLINE** | ("Metabolic Syndrome" [MeSH Terms] OR "Metabolic Syndrome" [All Fields] OR "insulin resistance" [All Fields] OR "cardiometabolic syndrome" [All Fields]) AND ("Atherogenic Index of Plasma" [All Fields] OR "AIP" [All Fields] OR "log(TG/HDL-C)" [All Fields]) AND ("cardiovascular disease" [MeSH Terms] OR "cardiovascular risk" [All Fields] OR "MACE" [All Fields] OR "coronary heart disease" [All Fields] OR "stroke" [All Fields] OR "mortality" [All Fields]). |
| **Scopus** | TITLE-ABS-KEY ( ( "metabolic syndrome" OR "insulin resistance" OR "cardiometabolic syndrome" OR "cardiovascular-kidney-metabolic" OR "CKM syndrome" OR "type 2 diabetes mellitus" OR "diabetic kidney disease" ) AND ( "atherogenic index of plasma" OR "AIP" OR "log(TG/HDL-C)" OR "atherogenic index" ) AND ( "cardiovascular disease" OR "cardiovascular risk" OR "MACE" OR "major adverse cardiovascular events" OR "coronary heart disease" OR "stroke" OR "mortality" OR "all-cause mortality" OR "cardiovascular mortality" ) ) |
| **Embase** | ('metabolic syndrome'/exp OR 'metabolic syndrome' OR 'insulin resistance' OR 'cardiometabolic syndrome' OR 'cardiovascular-kidney-metabolic syndrome' OR 'diabetic kidney disease') AND ('atherogenic index of plasma' OR 'AIP' OR 'log(TG/HDL-C)' OR 'atherogenic index') AND ('cardiovascular disease'/exp OR 'cardiovascular risk' OR 'major adverse cardiovascular events' OR 'MACE' OR 'coronary heart disease' OR 'stroke'/exp OR 'mortality'/exp OR 'all-cause mortality' OR 'cardiovascular mortality') |
| **Web of Science (WOS)** | TS=( ("metabolic syndrome" OR "insulin resistance" OR "cardiometabolic syndrome" OR "cardiovascular-kidney-metabolic" OR "CKM syndrome" OR "type 2 diabetes mellitus" OR "diabetic kidney disease") AND ("atherogenic index of plasma" OR "AIP" OR "log(TG/HDL-C)" OR "atherogenic index") AND ("cardiovascular disease" OR "cardiovascular risk" OR "MACE" OR "major adverse cardiovascular events" OR "coronary heart disease" OR "stroke" OR "mortality" OR "all-cause mortality" OR "cardiovascular mortality") ) |
